# Control of the eIF4E activity: structural insights and pharmacological implications

**DOI:** 10.1007/s00018-021-03938-z

**Published:** 2021-09-19

**Authors:** Alice Romagnoli, Mattia D’Agostino, Chiara Ardiccioni, Cristina Maracci, Stefano Motta, Anna La Teana, Daniele Di Marino

**Affiliations:** 1 Department of Life and Environmental Sciences, Polytechnic University of Marche, Via Brecce Bianche, 60131 Ancona, Italy; 2 New York-Marche Structural Biology Center (NY-MaSBiC), Polytechnic University of Marche, Ancona, Italy; 3 Department of Earth and Environmental Sciences, University of Milano-Bicocca, Milan, Italy

**Keywords:** eIF4E, Translation initiation, 4E-binding proteins (4E-BPs), Canonical eIF4E-binding motif, Non-canonical eIF4E-binding motif, Therapeutic target

## Abstract

The central role of eukaryotic translation initiation factor 4E (eIF4E) in controlling mRNA translation has been clearly assessed in the last decades. eIF4E function is essential for numerous physiological processes, such as protein synthesis, cellular growth and differentiation; dysregulation of its activity has been linked to ageing, cancer onset and progression and neurodevelopmental disorders, such as autism spectrum disorder (ASD) and Fragile X Syndrome (FXS). The interaction between eIF4E and the eukaryotic initiation factor 4G (eIF4G) is crucial for the assembly of the translational machinery, the initial step of mRNA translation. A well-characterized group of proteins, named 4E-binding proteins (4E-BPs), inhibits the eIF4E–eIF4G interaction by competing for the same binding site on the eIF4E surface. 4E-BPs and eIF4G share a single canonical motif for the interaction with a conserved hydrophobic patch of eIF4E. However, a second non-canonical and not conserved binding motif was recently detected for eIF4G and several 4E-BPs. Here, we review the structural features of the interaction between eIF4E and its molecular partners eIF4G and 4E-BPs, focusing on the implications of the recent structural and biochemical evidence for the development of new therapeutic strategies. The design of novel eIF4E-targeting molecules that inhibit translation might provide new avenues for the treatment of several conditions.

## Introduction

Translation initiation, the rate-limiting step in protein synthesis, is finely regulated by several mechanisms [1]. In the so-called “cap-dependent translation” the first step is represented by the assembly of the eukaryotic Initiation Factor 4F (eIF4F) complex and its binding to the mRNA 5´-cap, followed by the formation of the 43S preinitiation complex (43S PIC). The 43S PIC consists of the small ribosomal subunit 40S, eukaryotic translation initiation factors eIF1, eIF1A, eIF3, eIF5 and the eIF2–GTP–Met-tRNAi ternary complex [2]. The eIF4F complex then recruits the 43S PIC to the mRNA, leading to the formation of the 48S translation initiation complex (i.e., 48S IC). The 48S complex, whose structure has been recently resolved [3], scans the mRNA in the 5´–3´ direction until a start codon is found.

**Fig. 1:**
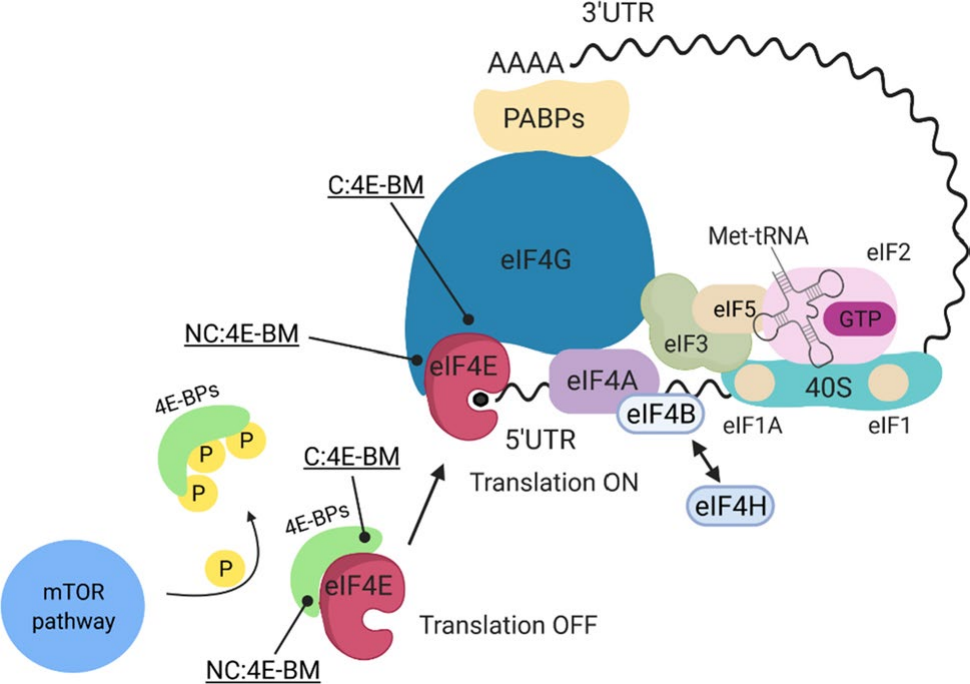
Schematic overview of protein synthesis and cap-dependent translation**.** The cap-binding factor eIF4E is released by 4E-BP as a result of its phosphorylation by mTOR. After the binding to the mRNA 5’-cap, eIF4E associates with the DEAD-box helicase Eukaryotic Initiation Factor 4A (eIF4A) and the Eukaryotic Initiation Factor 4G (eIF4G) to form the eIF4F complex. The interaction between eIF4G with the poly(A) binding proteins PABPs forms a closed-loop structure between 5’-UTR and 3’UTR. The ternary complex, consists of eIF2/GTP and met-tRNAi, that associates with the 40S, is also reported

eIF4F is an heterotrimeric complex composed by eIF4E, the DEAD-box helicase eIF4A and eIF4G [4] (Fig. 1). eIF4F has a peculiar assembly in which eIF4E binds the 7-methylguanosine (7-m-GTP) cap, whereas eIF4A unwinds the secondary structures in the 5´ untranslated regions (UTR) of the mRNAs. The scaffold protein eIF4G, besides its activity in recruiting the 43S PIC through the interaction with eIF3, promotes the circularization of mRNAs by interacting with the poly-A binding proteins (PABPs) (Fig. 1). eIF4E represents the limiting factor in the eIF4F complex, and therefore, it plays a pivotal role in the regulation of translation initiation rates. eIF4E is, in fact, one of the least abundant translation factors [5, 6] and its availability is regulated at multiple levels: transcriptionally, post-transcriptionally, post-translationally and through the interaction with a group of molecular partners exerting an inhibitory effect, namely, the 4E-binding proteins (4E-BPs) [7].

4E-BPs are a family of inhibitory proteins that sequester eIF4E through the interaction with the same binding site recognized by eIF4G, therefore, preventing the assembly of the eIF4F complex [8–10]. 4E-BPs, in turn, are the target of several kinases [11–13]; their phosphorylation causes the release of eIF4E and, consequently, a stimulation in translation initiation. Based on their dependence on eIF4E, mRNAs have been classified as “eIF4E-sensitive mRNAs” or “weak mRNAs” and “strong mRNAs”. The latter are typically transcripts of house-keeping genes, such as glyceraldehyde-3-phosphate dehydrogenase and ß-actin, characterized by short and unstructured 5´-UTRs. They show constitutive levels of translation and are scarcely influenced by eIF4E variations [14–16]. “Weak mRNAs”, on the other hand, have long and G/C-rich 5´-UTRs, able to form stable secondary structures [15], or 5´UTR containing oligopyrimidines sequences [17–19]. Translation of these mRNAs is strictly dependent on eIF4E. Several weak mRNAs code for proteins involved in cell survival and proliferation processes, such as cyclins D1 and D3, ornithine decarboxylase (ODC), vascular endothelial growth factor (VEGF), MYC and phosphoribosyl-pyrophosphatase synthetase 2 (PRPS2) [15, 16]. The tight control of eIF4E offers a mechanism to modulate translational rates in response to stress conditions, oncogenic stimulation, and changes of synaptic plasticity [20, 21]; variations in eIF4E levels have been implicated in neurodevelopmental and neuropsychiatric disorders, including ASD and FXS [22, 23], as well as in cancer; eIF4E has thus been defined as an oncogene [14, 15, 24].

In the last decades, the three-dimensional (3D) structure of eIF4E alone or in complex with its molecular partners from different organisms has been widely characterized using experimental and theoretical approaches [8, 10, 25–35].

**Fig. 2:**
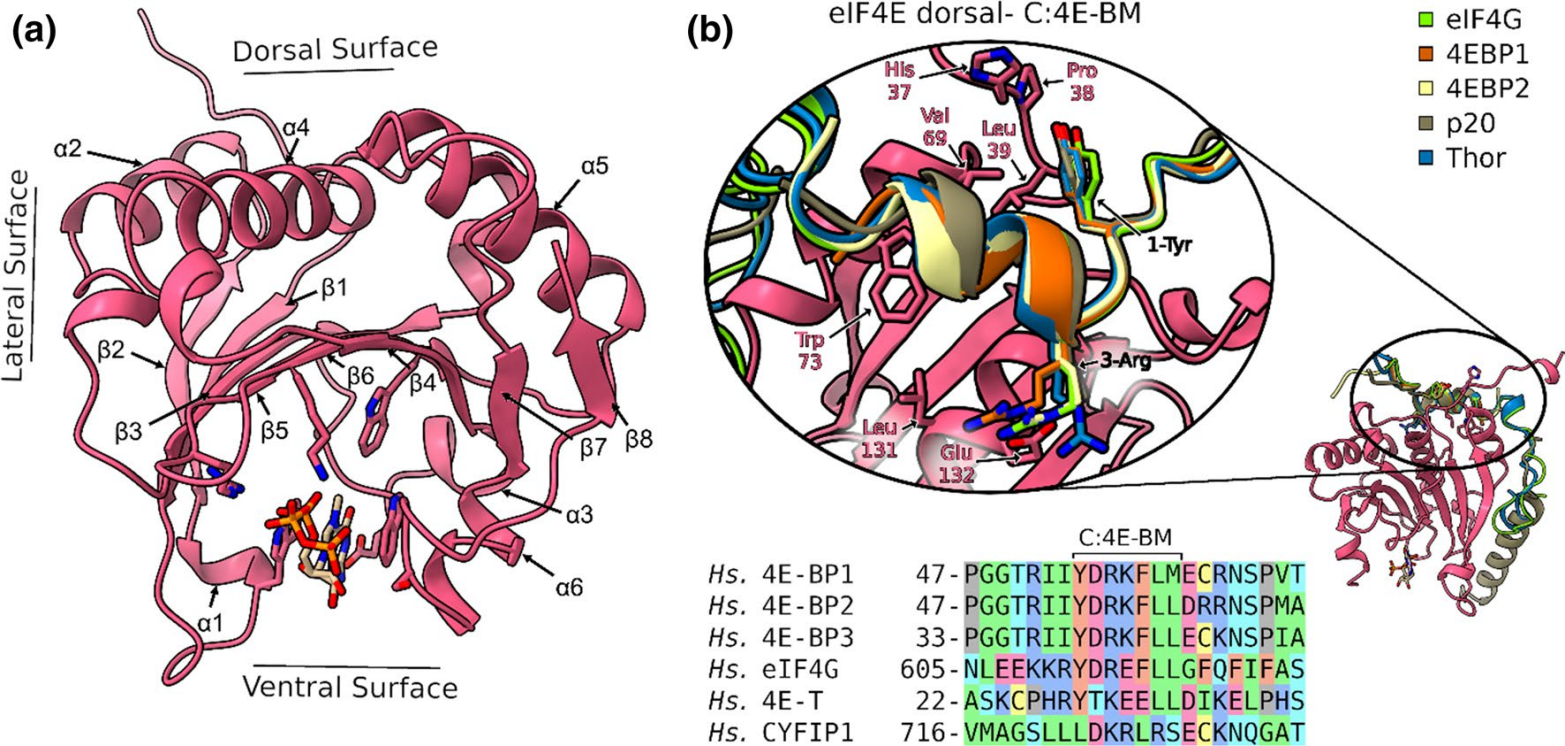
Overview of the *Hs* eIF4E structure (PDBID: 1IPC [30]) and structural details of C:4E-BM. **a** Cap-binding pocket situated on the ventral surface, together with dorsal and lateral surfaces, both responsible for the bipartite binding mode of 4E-BPs, are shown. The eIF4E residues involved in the 7 m-GTP cap binding are shown in sticks (see text); **b** top: Superimposition of the *Hs* eIF4G (PDBID: 5T46 [8]) and different 4E-BPs helices in the canonical site to highlight common structural features of eIF4G/4E-BPs binding mode with eIF4E (PDBID *Hs* 4E-BP1: 3U7X [178], PDBID *Hs* 4E-BP2: 3AM7, PDBID *Dm* THOR: 4UE8 [10], PDBID *Sc* P20: 6FC3 [10]); bottom: multiple sequence alignment of various *Hs* 4E-BM

Briefly, three main regions can be identified in the 3D structure of eIF4E (Fig. 2a): a ventral surface, where the cap-binding pocket is located, a dorsal surface and a lateral surface, both responsible for the binding with eIF4G and 4E-BPs through the canonical and the non-canonical eIF4E binding motifs, respectively [8, 29]. The advances in the structural knowledge opened new possibilities for the development of inhibitors acting on the translation initiation complex with high specificity and efficiency.

This review provides an overview of the roles of eIF4E and 4E-BPs in physiological and pathological conditions. We focus on the structural features of the interactions between eIF4E and different 4E-BPs and discuss new therapeutic strategies targeting eIF4E.

## eIF4E and 4E-BPs in health and disease

The main function of eIF4E is to mediate ribosome recruitment on the mRNA to start protein synthesis. Nevertheless, other roles for this protein have been described. eIF4E strongly enhances the helicase activity of eIF4A, independently of its cap-binding function [36]. The nuclear localization of eIF4E is needed for the control of nucleus-cytoplasmatic trafficking of some mRNAs harboring 7-m-GTP and a 50-nucleotides sequence at the 3´-UTR, defined “eIF4E sensitivity element” (4E-SE) [37]. eIF4E and these types of RNAs form “ribonucleoparticles” (RNPs) that translocate from nucleus to cytoplasm. Nuclear eIF4E was found to be associated with U1 small nuclear RNA, which participates in mRNA splicing [38], therefore, suggesting other nuclear functions for eIF4E.

eIF4E levels and activity are regulated by distinct mechanisms that involve pre- and post-transcriptional factors, post-translational modifications pathways and interactions with a subset of specific proteins [15]. Regulation at the transcriptional level is mediated by transcription factors, such as Myc, which activate *eif4e* transcription via binding to the E-boxes located in the eIF4E promoter [39]. Regulation at the post-transcriptional level is mediated by the HuR RNA-binding protein, which interacts with eIF4E mRNA, stabilizing and protecting it from degradation [40]. eIF4E was shown to be ubiquitinated at Lys 159 [41] and phosphorylated at Ser 209. The phosphorylation of Ser 209 is mediated by the activation of the MAPK/ERK pathway. The mitogen-activated protein kinase (MAPK)-interacting kinases (MNK1 and MNK2) bind the eIF4F complex, through the interaction with the C-terminal domain of eIF4G and phosphorylate Ser 209 of eIF4E [15, 42]. The biological significance of this post-translational modification is still debated [14, 43]. eIF4E phosphorylation does not seem to be essential for its function under physiological conditions [14, 44, 45]. Conversely, several studies suggest that the phosphorylation of eIF4E by MNK1 and MNK2, in response to stimuli, such as stress or mitogens, has direct effects on cancer onset and progression [17, 46–50].

In human cells, the overexpression or the dysregulation of eIF4E is the cause of a rapid development of lung, bladder, colon, prostate, breast, head and neck cancer [15, 17, 24, 49, 51].

As already mentioned, the activity and availability of eIF4E are regulated by the direct interaction with 4E-BPs. Three groups of eIF4E-interacting proteins have been identified: (i) proteins containing the Canonical eIF4E-Binding Motif (C:4E-BM): YXXXXLΦ, where X is any amino acid and Φ is a hydrophobic residue (Fig. 2b) [8]; (ii) proteins with the Really Interesting New Gene (RING) domain (as the promyelocytic leukemia protein and the arenaviral Z proteins) [52–55]; (iii) a small group of viral proteins that interact through a non-conserved motif [56–58].

These 4E-BPs compete with eIF4G for the interaction with eIF4E [8, 9, 31], therefore, acting as translational repressors by inhibiting the cap-dependent translation initiation. eIF4G and 4E-BPs share the C:4E-BM and thus the competition for the interaction with eIF4E is mainly due to a structural factor. The C:4E-BM interacts with a specific portion of eIF4E located on its dorsal surface, making the interaction with eIF4G or 4E-BPs mutually exclusive. From a structural point of view, the C:4E-BM folds in an α-helix structure interacting with several conserved hydrophobic amino acids on the dorsal surface of eIF4E, located at the opposite side with respect to the cap-binding pocket [8, 9, 29]. It has been recently reported that additional sequences, located C-terminally with respect to the C:4E-BM, play a key role in the interaction between eIF4E and 4E-BPs. These sequences include: (i) a non-canonical motif (NC:4E-BM) that binds to the lateral surface of eIF4E; (ii) a linker region which connects the two binding motifs (i.e., canonical and non-canonical) [8, 59–61]. Despite its poor sequence conservation, the NC:4E-BM motif, together with the linker region, increases the affinity for eIF4E and confers a competitive advantage compared to eIF4G [59, 62–68].

eIF4E activity is also regulated indirectly by the phosphorylation of the 4E-BPs as a direct response to extracellular stimuli, such as growth factors, oxygen shortage, nutrients availability, genotoxic stress, and inflammation [69]. In mammals three 4E-BPs have been identified: 4E-BP1, 4E-BP2, 4E-BP3, which are functionally similar but with different cellular localization. 4E-BP1 is the predominant eIF4E binding protein expressed in the majority of tissues, mostly in adipose tissues and in the pancreas [70]. Dysregulation of 4E-BP1 and mTOR pathway is associated with pathological states. Overexpression of 4E-BP1 is a crucial element in a specific subset of tumors, such as lung, prostate, breast and leukemia [71]. However, the pattern of 4E-BP1 expression and its role in cancer is not completely understood so far. As an inhibitor of the oncoprotein eIF4E, it is not surprising that 4E-BP1 acts as a suppressor of tumorigenesis; indeed, in vitro studies reported that 4E-BP1 is able to decrease cell invasion and migration in prostate and colon cancer [72, 73]. However, other studies demonstrated that 4E-BP1 activity is correlated to the tumor stage, development and progression, in particular in breast cancer [71]. Thus, 4E-BP1 and its phosphorylation might be used as a prognostic marker of cancer malignancy [15, 71].

4E-BP2 is the 4E binding protein preferentially expressed in the brain [74]. Alteration of cap-dependent protein translation has been implicated in ASD and FXS [4, 22]. As reported in several studies, 4E-BP2 knock-down mice display autistic-like behaviors [23, 74, 75]. More specifically, the deletion of 4E-BP2 in GABAergic inhibitory neurons resulted in altered social interaction and communication [75]. 4E-BP1 and 4E-BP2 are also involved in metabolic diseases, as double-knockout mice show the onset of obesity and insulin resistance [76].

The function and regulation of 4E-BP3, which is mostly expressed in the liver [77], is still poorly understood. 4E-BP3 works as a negative control of the transcription of “weak mRNAs”, but, differentially from the paralogues 4E-BP1 and 4E-BP2, its function is not regulated by phosphorylation [78].

The Cytoplasmatic FMRP Interacting Protein 1 (CYFIP1), another member of the 4E-BP family, has been extensively characterized, showing a specific role in the central nervous system [79]. CYFIP1 is also a binding partner of the Fragile X Mental Retardation Protein (FMRP), the protein absent or mutated in FXS [80, 81], suggesting a link between translational control and neurodevelopmental disorders [26, 27, 79, 80, 82]. The expansion of a trinucleotide CGG repeat upstream of *FMR1 gene* leads to FXS pathology, an X-linked inherited intellectual disability [83]. Lack of FMRP causes abnormal translation of specific transcripts at the synapse during critical stages of neurodevelopment [84]. CYFIP1 forms a trimeric complex with FMRP and eIF4E [79, 82], thereby repressing the translation of some key mRNAs in neurons that lead to aberrant morphology of dendritic spines (i.e., the molecular phenotype of FXS) having a direct effect on the correct synapses formation [79]. Furthermore, CYFIP1 is a so-called moonlighting protein, because it plays a central role in two separate pathways, thanks to its ability to acquire two distinct structural conformations. Indeed, CYFIP1 is a component of the WAVE regulatory complex (WRC), a hetero-pentameric complex essential for controlling actin polymerization in the cell [82, 85]. Synaptic activation by Brain-derived neurotrophic factor (BDNF) or group I metabotropic glutamate receptors (mGluRs) causes the dissociation of CYFIP1 from eIF4E, triggering the cap-dependent translation initiation, and consequent recruitment of CYFIP1 toward the WAVE complex [79, 82]. A correct balance between translation initiation complex and WAVE is crucial for spine morphogenesis in neurons, synapses development and neurons functionality. CYFIP1 is in fact involved in other neurological disorders, such as schizophrenia (SCZ) and autism [22]. In particular, CYFIP1-deficient mice display defects in the structure of *corpus callosum*, which leads to alteration in brain functional connectivity, sensory perception and coordination, typical tracts of neuropsychiatric diseases [22, 86]. CYFIP1 is also involved in tumorigenesis, as it is considered a tumor suppressor gene [87–89].

The list of proteins that specifically bind to eIF4E is not restricted to those mentioned so far but includes many other proteins, some of them share the characteristic of regulating a specific subset of mRNAs. Some of them are shared by several taxa, such as 4E-T, Maskin and Neuroguidin [90]. Human 4E-T was initially identified as a cytoplasmatic shuttle protein that translocates eIF4E to the nucleus but, more recently, a cytoplasmatic function of 4E-T in stimulating P-bodies formation [91–93] and in mRNA decay was also described [94], indicating that it modulates the expression of crucial genes involved in neurogenesis and oogenesis [95, 96]. A short segment of 4E-T shares limited homology with the *D. melanogaster* CUP protein [91]. This 4E-BP is involved in *D. melanogaster* embryogenesis, since it impairs the eIF4F complex formation and thus specifically represses the translation of *oskar*, *nanos* and *gurken* mRNAs, which are essential for development. CUP is able to regulate specific target mRNAs working as a molecular adaptor and interacting with other RNA-binding proteins, able to recognize specific sequences or structural elements located at the 3′UTR of these mRNAs, such as Bruno, which binds *oskar* mRNA, and Smaug, which binds *nanos* mRNA [63, 97–102]. Maskin, another 4E-binding partner belonging to this group, blocks translation of specific mRNAs during *X. laevis* oocytes development by simultaneously binding to eIF4E and CPEB (cytoplasmic polyadenylation element binding protein). The latter, in turn, recognizes all mRNAs bearing a CPE (cytoplasmic polyadenylation element) in their 3′-UTR. Assembly of this complex, also including other proteins, causes the shortening of the mRNAs polyA tails making them untranslatable. During development, activation of their translation is triggered by specific signals which induces the complex disassembly and the mRNA polyA-tail elongation. [97, 98]. A 4E-BP and CPEB protein that acts similarly is Neuroguidin, a translation repressor of mRNAs containing the CPE element, found in all eukaryotic lineages but studied in particular in neuronal cells during *X. laevis* neurogenesis [90]. Other proteins interacting with the initiation factor 4E are: the RNA helicase DDX3, that promotes survival in stressed cells through its interaction with eIF4E [103]; the CCR4 deadenylase family member Angel1; the RNA-binding protein Gem-associated protein 5 GEMIN-5 [104–106]; a group of homeodomain proteins (i.e., HoxA9, Hox11, Emx2, Otx2 and Engrailed 2) [107–109], the 4E-BP1 ortholog protein Thor in *D. melanogaster*; the unique lineage-specific 4E-BPs in yeast: p20 and eIF4E-associated protein 1 (Eap1p); and the unusual 4E-BP Mextli [10, 61, 68, 110–115]. Despite the different function, origin and sequence of all these 4E-BPs, they display common structural features concerning the interaction with eIF4E, that will be punctually described in the next chapters. An extensive list of the best functionally or structurally characterized 4E-BPs is summarized in Table 1.

**Table 1:** Most of the functionally or structurally characterized 4E-BPs in human and in other organisms

Name of 4E-BP	UNIPROT code	PDB code	Organism	Pathological implication
4E-BP1	Q13541	1WKW,6BCX, 3U7X 4UED, 6BCU, 6BCX	*H. sapiens*	Tumorigenesis and metabolic diseases [75, 116]
4E-BP2	Q13542	2MX4, 3AM7	*H. sapiens*	Neuropsychiatric disorders and metabolic syndromes [73–75]
4E-BP3	O60516		*H. sapiens*	
CYFIP1	Q7L576	3P8C, 4N78	*H. sapiens*	Tumorigenesis; neuropsychiatric and neurodevelopmental disorders [78, 79, 86, 89]
4E-T	Q9NRA8	5ANR, 6F9W	*H. sapiens*	
ANGEL1	Q9UNK9		*H. sapiens*	
DDX3X	O00571	6CZ5, 5E7I, 4PX9, 2JGN, 2I4I	*H. sapiens*	Tumorigenesis and neurodevelopmental disorders [117–119]
GEMIN-5	Q8TEQ6	5GXH, 5GXI, 5h1J, 5H1K, 5H1L, 5H1M, 5H3S, 5H3T, 5H3U, 5TEE, 5TEF, 5THA, 6RNQ, 6RNS	*H. sapiens*	
4E-T	Q8IH18	4UE9	*D. melanogaster*	
CUP	Q9VMA3	4AXG	*D. melanogaster*	Development [62]
THOR	Q9XZ56	4UE8	*D. melanogaster*	Innate immunity, cell growth, synaptic transmission [120–122]
MEXTLI	Q9VR35	5ABV	*D. melanogaster*	Germline stem cell maintenance and early embryogenesis [115]
MASKIN	Q9PTG8		*X. laevis*	Development [97, 98]
NEUROGUIDIN	Q4KLC4		*X. laevis*	Neurogenesis [90]
P20	P12962	6FC3	*S. cerevisiae*	
EAP1P	P36041	6FC2	*S. cerevisiae*	
MEXTLI	Q9XW13	5ABY	*C. elegans*	
Homeodomain proteins (BICOID, HoxA9, Hox11, Emx2, Otx2 and Engrailed 2)		1ZQ3, 1PUF, 2DMS, 3ZOB		Neurogenesis [109]

### Control of protein synthesis by phosphorylation of 4E-BPs

The activity of the 4E-BPs and the ability to interact with eIF4E, as mentioned above, is regulated by their phosphorylation. The main enzyme involved is the serine/threonine kinase mammalian target of rapamycin (mTOR) [11]. The mTOR pathway is activated by PI3K-Akt (protein kinase B); activation of the mTOR complex 1 leads to phosphorylation of 4E-BPs at multiple sites (Thr 37, Thr 46, Ser 65, Thr 70), causing the release of eIF4E (reviewed in [17]). The higher level of eIF4E in the cell leads to an increase of the cap-dependent translation, thus affecting the translation of “weak mRNAs”, highly dependent on the eIF4E activation state [1, 49, 123, 124]. Notably, dysregulation of the eIF4E/4E-BPs interaction is a common feature of numerous diseases, such as cancer, neuropsychiatric and neurodevelopmental disorders [22, 125].

4E-BPs have multiple phosphorylation sites depending on the isoform or species [13]. In 4E-BP1, seven phosphorylation sites have been identified: Thr 37, Thr 46, Ser 65, Thr 70, Ser 83, Ser 101, and Ser 112 (*Hs* numbering). The first five phosphorylation sites are conserved among 4E-BPs, whereas Ser 101 and Ser 112 are peculiar of 4E-BP1 [126, 127]; furthermore, the kinase(s) responsible for Ser 101 and Ser 112 phosphorylation has(have) not been identified yet. Albeit conserved among mammalian 4E-BPs, Ser 83 seems to have a minor impact in the control of translation initiation [10]. Moreover, two phosphorylation sites are situated on the linker regions of the downregulating 4E-BP (Ser 65 and Thr 70 on *Dm* Thor).

Phosphorylation of 4E-BPs by mTOR follows a stepwise mechanism [12, 13, 126, 127]; first, T37 and T46, are phosphorylated, resulting in a hypo-phosphorylated 4E-BPs state [128]. Phosphorylation of the early sites triggers the folding of residues Pro 18–Arg 62 (4E-BP2, PDBID 2MX4) into a four-stranded ß-domain, sequestering the C:4E-BM into a partially covered ß-strand and consequently obstructing its accessibility to eIF4E. This folded and partially phosphorylated state is less stable and causes a decrease in the binding affinity for eIF4E by 100-fold, but still being able to inhibit eIF4G binding [129]. Subsequent phosphorylation of Ser 65, Thr 70 and Ser 83, located in the C-terminal intrinsically disordered region (C-IDR) of 4E-BPs, stabilizes the β-folded domain conformation, which is incompatible with eIF4E binding [130]. Fully phosphorylated 4E-BPs show a 4000-fold decreased affinity for eIF4E.

**Fig. 3:**
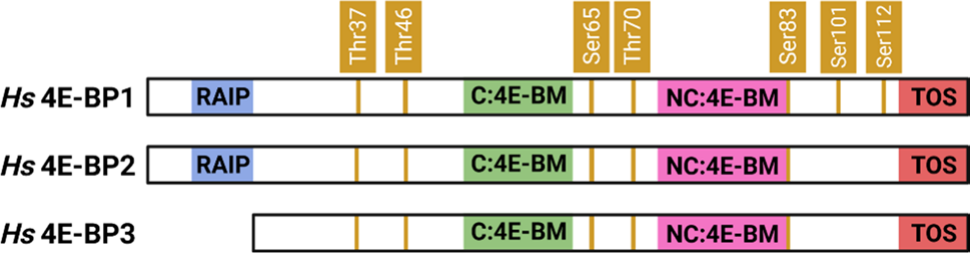
Schematic illustration of the primary structures of the three human 4E-BPs. The Threonine (Thr) and Serine (Ser) residues that undergo phosphorylation are numbered for 4E-BP1 (dark orange) [126]. The C:4E-BM (green) and NC:4E-BM (pink), together with RAIP (light blue) [131] and TOS (red) [127] motifs are shown

The interaction of 4E-BPs with mTOR is mediated by the adaptor protein Raptor, an essential component of mTORC1 [131, 132]. Two short motifs are responsible for Raptor binding and 4E-BP tethering: the mTOR signalling (TOS) motif, which serves as a docking site for Raptor, and the RAIP motif (i.e., Arg-Ala-Ile-Pro) named after its sequence, which is located at the N-terminus of 4E-BP1 and 4E-BP2, but is absent in 4E-BP3 [133, 134] (Fig. 3). The tethering of 4E-BP1 by both motifs reduces the conformational entropy of the protein, which is then poised for phosphorylation. Moreover, the interaction between 4E-BP1 and Raptor seems to be independent of the presence of eIF4E, which makes phosphorylation of the early sites (Thr 37 and Thr 46) highly efficient [135].

## Structural insights into the 4E-BPs canonical binding motif

The three-dimensional structure of eIF4E from many organisms has been solved, allowing an accurate structural comparison among species [28–32]. The overall fold of eIF4E is extremely conserved, adopting a horseshoe-like conformation characterized by the presence of a ß-sheet containing eight antiparallel ß-strands, three long α-helices (α2, α4 and α5), which cover the convex hydrophobic face of the ß-sheet and form the dorsal binding site of the protein, and three short α-helices (α1, α3 and α6), oriented perpendicularly to the plane of the ß-sheet, primarily constituting the concave/ventral surface, opposite to the distal side of the protein [28, 29] (Fig. 2a). The cap-binding site is located in the ventral surface of the protein, about 25 Å far from the distal side. The interaction between eIF4E and the cap is mostly determined by the formation of cation-п stacking interactions between the 7-methylguanine and two conserved tryptophan residues in the cap-binding pocket (Trp 56 and Trp 102, PDBID: 1IPC) (Fig. 2a). This interaction is also stabilized by a hydrogen bond network involving a conserved glutamate residue (Glu 103), the N1 and N2 atoms of the 7-methylguanine [30, 136] and positively charged residues (Arg 157, Lys 159 and Lys 162) located in the β5–β6 loop, that interact with the α- and β-phosphate oxygen atoms of the cap (Fig. 2), a key interaction for the phosphate binding. The dorsal surface of eIF4E shows an invariant hydrophobic/acidic area and is responsible for the binding with protein partners, including eIF4G and the 4E-BPs [28]. This interaction relies on the canonical eIF4E-binding site motif (C:4E-BM), located in eIF4G and 4E-BPs, of the consensus sequence YXXXXLΦ (where X is any residue and Φ is any hydrophobic amino acid) (Fig. 2b) that adopts a conserved α-helical fold [9, 28, 137], as shown in Fig. 2b. The interactions between eIF4E and the C:4E-BM are particularly conserved among different proteins: the hydroxyl group of the tyrosine side chain (*Hs* Tyr 612 or *Dm* Tyr 621) forms a hydrogen bond with the carboxyl oxygen of the proline within the backbone of the His-Pro-Leu conserved motif in elF4E (*Hs* His 37–Pro 38–Leu 39 or *Dm* His 70–Pro 71–Leu 72) and establishes van der Waals interactions with a valine of eIF4E (*Hs* Val 69 or *Dm* Val 102). Moreover, the conserved residues (*Hs* Val 69, Trp 73 and Leu 131) located on the dorsal surface of eIF4E are in contact with the hydrophobic amino acids of C:4E-BM (LΦ) at the C-terminus (Fig. 2b). The majority of the 4E-BPs and metazoan eIF4Gs have the consensus motif that includes aliphatic amino acids (R/K/Q) at the positions 3 and 10, bringing to an extended canonical binding sequence: YX(R/K)X_2_LΦX_2_(R/K/Q). These residues contribute to the binding with eIF4E, most probably by covering hydrophobic surface areas of eIF4E from the exposure to solvent [8, 10, 25, 35]. Moreover, the arginine/lysine located at the position 3 stabilizes the interaction of the canonical helix, making a salt bridge with the conserved Glu 132 in eIF4E, conserved in metazoans.

Among the 4E-BPs, CYFIP1 is atypical: it bears a sequence variation of the canonical C:4E-BM, with a leucine instead of tyrosine at position 1, arginine instead of leucine at position 6, while the hydrophobic amino acid at position 7 of the canonical site (i.e., LDKRLRS) is absent [79]. The variation of the C:4E-BM of CYFIP1 brings to a new network of amino acids interactions with eIF4E, as a peptide extracted from the eIF4E-binding domain of CYFIP1 (i.e., CYFIP1p: residues 721–734) acquires a new orientation within the canonical binding site portion [26], forming a peculiar “reverse L shaped” structure [79]. The crystal structure of CYFIP1/eIF4E is not currently available, but important structural information have been obtained from molecular dynamic simulations performed on CYFIP1, which point out to a unique binding mode with eIF4E, compared with eIF4G and 4E-BPs (Fig. 2b) [26]. This structural prediction is also consistent with in vitro experiments previously performed on several CYFIP1 mutants [79].

Additional sequences situated at the C-terminus of the C:4E-BM are involved in the association of 4E-BPs with eIF4E; they include a linker region and a Non-Canonical Binding Motif (NC:4E-BM) and increase the affinity of 4E-BPs for eIF4E by 2 to 3 orders of magnitude [8, 10, 59, 64–67].

### Structural insights into the 4E-BPs non-canonical binding motif

**Fig. 4:**
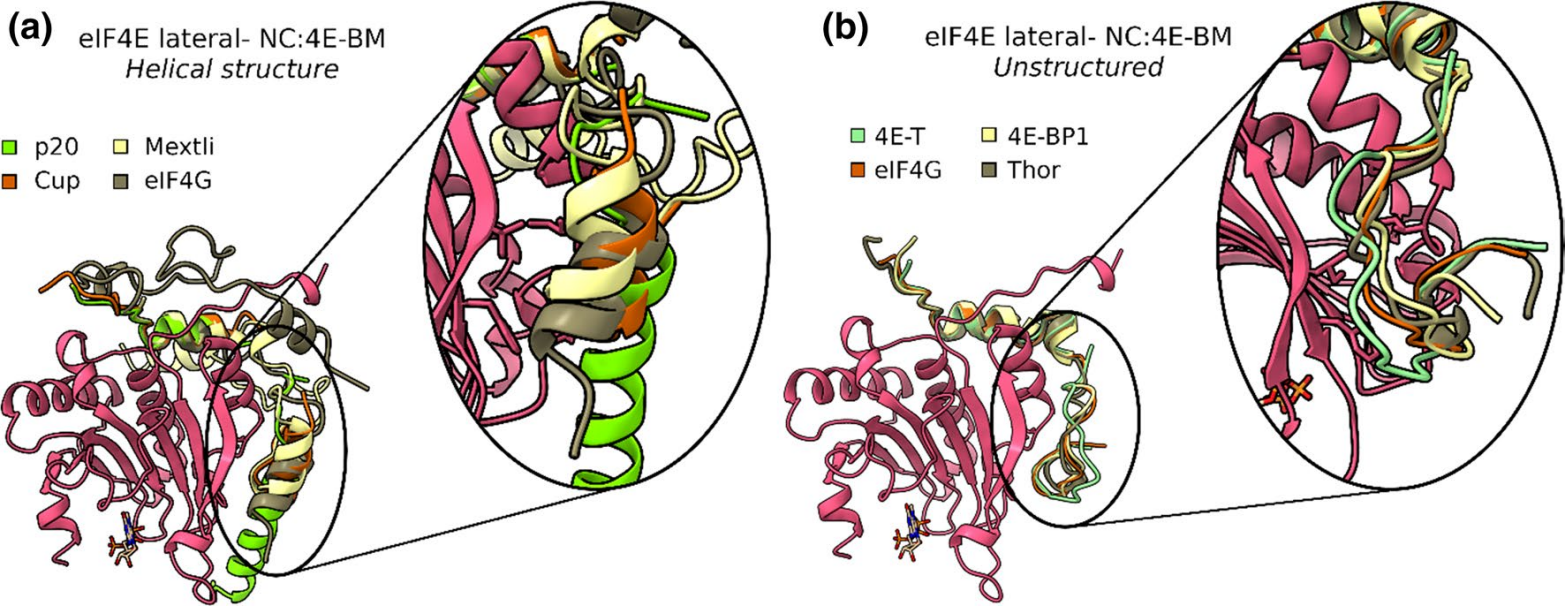
Lateral surface of eIF4E in complex with different NC:4E-BM of 4E-BPs. **a** Superimposition of the secondary structure of eIF4E bound to *Ct* eIF4G (PDBID: 6FC0 [61]), *Dm* MEXTLI (PDBID: 5ABV [68]), *Sc* p20 (PDBID: 6FC3 [10]), with the non-canonical binding motif structured in α-helix; **b** superimposition of the secondary structure of the eIF4E bound to *Hs* eIF4G (PDBID: 5T46 [8]), *Dm* 4E-T (PDBID: 4UE9 [10]), *Dm* THOR (PDBID: 4UE8 [10]), with the unstructured non-canonical binding motif

**Fig. 5:**
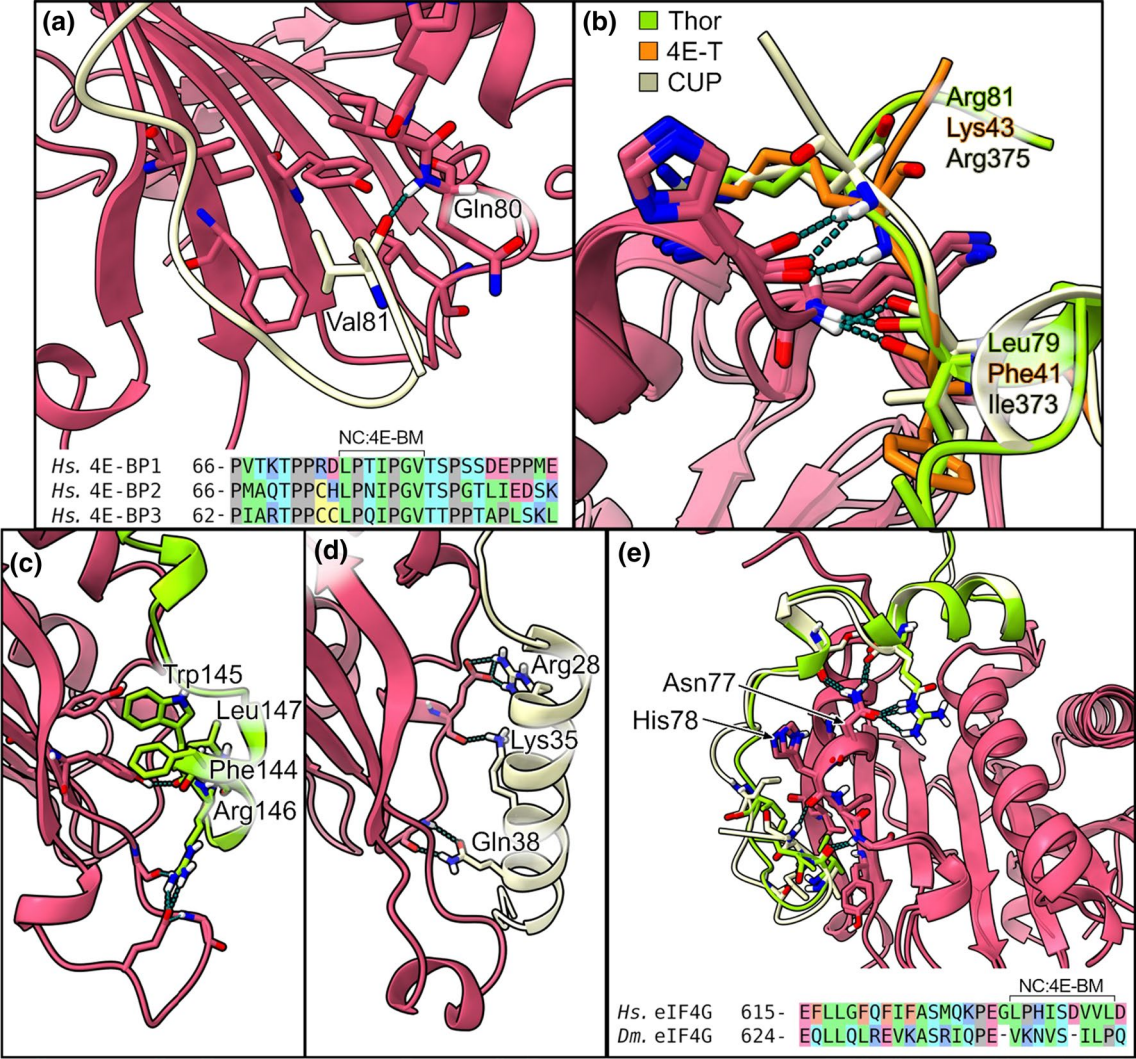
Molecular details of the non-canonical binding sites in different eIF4E complexes. **a** Close-up view of the non-canonical binding site of *Hs* 4E-BP1 (PDBID: 1WKW [179]) and sequence alignment of the homologous *Hs* 4E-BP2 and *Hs* 4E-BP3; **b** interactions in the non-canonical binding site of Thor (PDBID: 4UE8 [10]), 4E-T (PDBID: 4UE9 [10]) and CUP (PDBID: 4AXG [63]); **c**, **d** close-up views of p20 (PDBID: 6FC3 [61]) (**c**) and Ea1p1 (PDBID: 6FC2 [138]) (**d**) that form helices in the NC:4E-BM; **e** comparison of the *Hs* (PDBID: 5T46 [8]) and *Dm* (PDBID: 5T47 [8]) eIF4G complexes

Although the eIF4E C:4E-BM was reported for several years as the main binding region for eIF4E [137], the recently described NC:4E-BM plays an equally important role in the regulation of cap-dependent translation [62]. Pivotal work by the group of Elisa Izaurralde has shown that the competition between eIF4G and 4E-BPs is strictly related to the NC:4E-BM, which is, however, poorly conserved among 4E-BPs [10]. In particular, the structural characterization of C:4E-BM and NC:4E-BM combined with several kinetic measurements has unveiled the relation between binding mechanism and affinity for eIF4E. Despite the high sequence variability of the NC:4E-BM, it recognizes the same hydrophobic pocket on the lateral surface of eIF4E (Fig. 4). The lateral binding site of eIF4E is composed of a group of well-conserved amino acids: Phe 47, Ile 63, and Ile 79 in *Homo sapiens* (Tyr 80, Ile 96, and Ile 112 in *Drosophila melanogaster* (*Dm*) eIF4E) [10]. Importantly, this region is approached by various 4E-BPs with very different backbone conformations; only a few have a well-defined secondary structure (Fig. 4a). Interestingly, the non-canonical motifs of *Dm* Mextli (PDBID: 5ABV), *Caenorhabditis elegans* (*Ce*) Mextli (PDBID: 5ABY), *Dm* CUP (PDBID: 4AXG), *Saccharomyces cerevisiae* (*Sc*) p20 (PDBID: 6FC3), *Sc* Eap1p (PDBID: 6FC2) and *Chaetomium thermophilum* (*Ct*) eIF4G (PDBID: 6FC0) display a helical structure [10, 61, 68] (Fig. 4a). Notably, *Dm* Mextli (PDBID: 5ABV) contains a C-terminal auxiliary helix that provides an unusual tripartite binding mode with eIF4E [68]. Human 4E-BP1 (PDBID: 4UED) and its subtypes (4E-BP2, 4E-BP3) regulate protein synthesis via competition with eIF4G for binding to eIF4E and they use for this interaction both canonical and non-canonical motifs, this being a crucial feature for the correct interaction with eIF4E and its regulation. Indeed, deletion of the C-terminal non-canonical region of 4E-BP1, where most of the essential interacting residues (79 Pro-Gly-Val-Thr-Ser 83) are situated, weakens the binding with eIF4E by 2 orders of magnitude. Within the NC:4E-BM sequence, Val 81 is the most important residue and interacts with the lateral surface of eIF4E through its hydrophobic side chain [66]. In addition, the nitrogen of eIF4E Gln 80 stabilizes the carbonyl oxygen of 4E-BP1 Val 81 through a main-chain contact [10]. The deletion of this single valine weakens the binding between 4E-BP1 (PDBID: 4UED) and eIF4E by an order of magnitude [66]. 4E-BP2 binds eIF4E in a dynamic way and it uses the usual bipartite mode [67] (Fig. 5a).

The binding of *D. melanogaster* 4E-BPs to eIF4E has been extensively characterized; CUP (PDBID: 4AXG), 4E-T (PDBID: 4UE9) and the 4E-BP1 ortholog protein Thor (PDBID: 4UE8) interact with eIF4E through the same bipartite mechanism involving both canonical and non-canonical motifs. The non-canonical regions interact with the hydrophobic pocket of eIF4E in a similar way to human proteins, with a crucial residue that engages the lateral surface of eIF4E by hydrophobic contact (Leu 79 in Thor, Phe 41 in 4E-T and Ile 373 in CUP). The carbonyl oxygens of these residues, as well as Val 81 in 4E-BP1, are fixed by a main-chain contact to the nitrogen atom of Lys 113 [*Dm* eIF4E]. Unlike 4E-BP1, these three proteins form an additional main-chain contact between the nitrogen atom of Arg 375 [CUP] (Lys 43 in 4E-Tand Arg 81 in Thor) and the carbonyl oxygens of His 111 [*Dm* 4E] [10, 63] (Fig. 5b). In yeast, p20 and Eap1p are deregulators of the cap-dependent translation exerting their function through competition with eIF4G for binding to eIF4E [113, 114]. Izaurralde and colleagues have recently determined the crystal structures of these two *Sc* 4E-BPs in complex with *Sc* eIF4E, confirming the conserved binding mode between the non-canonical site and the lateral surface, despite the poor sequence conservation [61]. Interestingly, p20 and Eap1p1 non-canonical motifs fold into an α-helix (Fig. 4a). Specifically, in p20 (PDBID: 6FC3) residues Ala 21-Lys 41 form a long amphipathic α-helix that establishes hydrophobic interactions over the lateral surface of eIF4E. In addition, some residues form salt bridges and hydrogen bonds with the lateral surface of eIF4E. Together, the hydrophobic and polar interactions of the amphipathic α-helix stabilize the overall binding between *Sc* p20 and *Sc* eIF4E (Fig. 5c). The α-helix formed by the non-canonical motif of Ea1p1 (PDBID: 6FC2) is shorter compared to the one of p20 (only 8 residues, Pro 130–Arg 137) and contributes less to the interaction with eIF4E via hydrophobic contacts. Furthermore, Ea1p1contains an N-terminal auxiliary extension that folds into a ‘hairpin’-like motif on the eIF4E dorsal surface and thus increases the buried surface area of the eIF4E–Eap1p interface. Notably, the resulting extensive interactions between eIF4E and Eap1p have not been described in any reported structure of 4E–BPs–eIF4E complexes [138]. However, a second unstructured region called FWRL motif (Phe 144–Leu 147) is found C-terminally to the α-helix. This motif contributes significantly to the interaction between Ea1p1 and the lateral surface of eIF4E via an extensive T-shaped π–π-stacking involving Trp 145 [*Sc* Ea1p1] at the center, Phe 144 [*Sc* Ea1p1], Tyr 47 [*Sc* eIF4E] and Tyr 93 [*Sc* eIF4E] [138]. Moreover, a salt bridge between the guanidinium group of Arg 146 [*Sc* Ea1p1] and the carboxyl moiety of Glu 56 [*Sc* eIF4E] contributes to the stability of the Eap1p–eIF4E interaction [138] (Fig. 5d).

eIF4G interacts with eIF4E in the same way as the other 4E-BPs. Structurally, the eIF4G surface deputed to interact with eIF4E is composed by the same three regions found in 4E-BPs (i.e., C:4E-BM, linker and NC:4E-BM). The linker region, that is located immediately after the C:4E-BM, forms an elbow loop that orients the NC:4E-BM towards the lateral surface of eIF4E and engages this area with hydrophobic interactions. Specifically, the *Hs* eIF4G (PDBID: 5T46) residues Leu 633, Ile 636, Val 639 and Val 640 interact with the hydrophobic residues Phe 47, Ile 63, Leu 75 and Ile 79of *Hs* eIF4E, while in *Dm* eIF4G (PDBID: 4UEC) the residues involved in the hydrophobic interaction are Val 641, Ile 646, Leu 647 that engage Tyr 80, Ile 96, Leu 108 and Ile 112, respectively, of *Dm* eIF4E. The linker region engages the surface of eIF4E through the interaction with two conserved eIF4E residues: *Hs* Asn 77 (or Dm Asn 110) and *Hs* His 78 (or *Dm* His 111) [8] (Fig. 5e).

In 2011 Umenaga et al*.* [139] have discovered the *Hs* eIF4G non-canonical binding site (572Tyr-Asp-Arg-Glu-Phe-Leu-Leu78) that, unlike the fungal *Chaetomium thermophilum* homologous protein, does not have a defined secondary structure (Fig. 4b) [8, 61]. This study allowed a better understanding of the competitive mechanism behind the regulation of cap-dependent translation. They have measured the kinetic parameters of the interaction between some 4E-BPs (*Hs* 4E-BP2, *Hs* 4E-BP1, *Dm* Thor) and *Hs* eIF4G fragment peptides with eIF4E, establishing that eIF4G non-canonical motif has a lower binding affinity than the non-canonical binding motif of 4E-BP2 for the cap-binding protein [8, 61]. This discrepancy is due to the different amino acid composition of both the linker region and the non-canonical binding motif. Interestingly, structure of *Saccharomyces cerevisiae* eIF4E–eIF4G complex (PDBID: 1RF8) [140] reveals a different interaction interface between the two proteins. The auxiliary flanking region of eIF4G, which might represent the non-canonical motif, does not interact with the lateral surface of eIF4E. This sequence folds into a bracelet-like structure that coats around the N-terminal region of eIF4E. Nevertheless, this discrepancy may be explained by the use of CHAPS, a zwitterionic detergent, during the preparation of the proteins for determination of the NMR structure [140]. CHAPS may have interfered with the hydrophobic interaction network present at the lateral surface of eIF4E. In this context, Gruner et al. support the theory that *Sc* eIF4G also binds *Sc* eIF4E through the conserved bipartite mechanism, although the crystal structure of the complex is not available yet [138]. They have performed small angle X-ray scattering (SAXS), homology modeling and pull-down assay to study the conformation and the physical interaction of the *Sc* eIF4G–eIF4E demonstrating that the complex likely adopts a conformation very similar to *Ct* eIF4E–eIF4G [138]. The three-dimensional structure of the fungal (*Chaetomium thermophilum*) eIF4G (PDBID: 6FC0) shows the conserved bipartite binding mechanism described for metazoan 4E-BPs but with some striking differences. In addition to the NC:4E-BM α-helix binding to the lateral surface of eIF4E, in the N-terminal segment two additional α-helices wrap around the N-terminus region of eIF4E forming a bracelet-like structure which significantly contributes to enlarge the *Ct* eIF4E–eIF4G interface, as observed only for *Sc* eIF4E–eIF4G complex [61]. In conclusion, the binding affinity of eIF4G and 4E-BPs for eIF4E is similar [8, 62], and what significantly provides an advantage to 4E-BPs rather than eIF4G in the interaction with eIF4E is indeed the linker and the NC:4E-BM [8], highlighting the key role of these regions, together with the phosphorylation sites, in regulating the cap-dependent translation.

## Targeting eIF4E: towards possible pharmacological applications

One of the main features of cancer onset and progression is the malfunctioning of the translation machinery, resulting in an increase of protein synthesis due to eIF4E dysregulation. eIF4E has thus been recognized as an attractive pharmacological target in particular for anticancer therapy. To date, several inhibitors targeting eIF4E have been developed. A subgroup of these inhibitors acts directly repressing the translation of eIF4E mRNA; others by blocking the interaction between eIF4E and its partners. Furthermore, indirect strategies implying the use of inhibitors that act on phosphorylation pathways have been also developed [14, 15]. Because eIF4E function is strictly dependent on its binding to the 7-m-GTP cap, molecules able to block or to compete with this interaction are becoming more and more attractive as drug candidates. One representative approach is the use of the cap-binding antagonists derived from 7-m-GTP analyzed for their ability to compete with eIF4E for the binding to capped mRNAs [141–143]. Several molecules extracted from 7-m-GTP analogues libraries have been selected for their favorable drug-like properties. Among them, 7-benzyl guanosine monophosphate (Bn7GMP) [144], and its derivative 4Ei-1 [145, 146], seem to be the most relevant one based on in vitro and in vivo studies [144–147]. Another direct strategy for targeting eIF4E overexpression is the use of specific antisense oligonucleotide (ASO) or small interference RNA (siRNA). ASOs are short (10–25 nucleotides) single-stranded DNA oligonucleotides that are able to target a specific mRNA through complementary hybridization [148]. Some eIF4E ASOs were designed also to recruit endogenous RNase H, therefore, decreasing eIF4E expression; studies performed using eIF4E ASOs on different cancer cell lines show promising results [149–152]. eIF4E knock-down by siRNA can arrest cell cycle, suppress cell mobility and colony formation in MDA–MB-231 triple-negative breast cancer cells [153]. Moreover, an eIF4E specific siRNA has been shown to inhibit cell growth in squamous carcinoma and adenocarcinoma [154, 155].

eIF4E is also indirectly targeted by inhibitors of the mTOR pathway, which interrupt the upstream signals for the phosphorylation of 4E-BPs, consequently preventing the dissociation between eIF4E and 4E-BPs. The best characterized compound is rapamycin (or sirolimus), a macrolide produced by *Streptomyces hygroscopicus* that is able to exert an allosteric inhibitory effect on mTORC1. Rapamycin has shown antineoplastic effects towards several cancer cell lines and mouse models [14]. However, several rapamycin analogues (rapalogues) with better pharmacological characteristics are already under clinical trials, such as CCI-799 (temsirolimus), AP23573 (deforolimus), RAD001 (everolimus), SAR943 (32-Deoxorapamycin), ABT-578 (zotarolimus) [14, 156–161]. Temsirolimus is FDA-approved in renal carcinoma [162–166], while everolimus is FDA-approved for neuroendocrine and breast cancers and deforolimus shows antitumor effects in hematologic tumors [14]. Other second-generation mTOR inhibitors have been developed and named active-site mTOR inhibitors (TORi). Among these, Torin 1, AZD8055, WYE-125132, INK128, PP242 have shown antitumor properties in vivo and in vitro, with enhanced bioavailability and efficacy compared to rapamycin [14, 15]. The administration of all these molecules, however, raised negative side effects, limiting their potential use [15]. Another promising pathway that can be targeted is the Mitogen-Activated Protein Kinase Interacting Protein Kinases (MNKs). Inhibitors, such as CGP57380, CGP052088 and cercosporamide, reduce the phosphorylation of eIF4E Ser 209 and showed anti-neoplastic features in cell cultures, especially in the treatment of metastatic cells. Nevertheless, also in this case the molecules show off-target problems [167].

Structural properties play a key role in the regulation of cap-dependent translation; targeting eIF4E/eIF4G interaction is thus another promising approach. High-throughput screening assays identified small-molecule inhibitors [168], such as 4EGI-1, 4E1RCat and 4E2RCat [169]. The X-ray structure of eIF4E-bound 4EGI-1 shows that 4EGI-1 blocks the binding of eIF4G interacting with a specific hydrophobic/basic pocket of eIF4E, and is thus considered an allosteric inhibitor [170]. Because it displays a dual activity, by inhibiting the eIF4G/eIF4E complex formation and enhancing the binding of 4E-BP1, 4EGI-1 is considered one of the most effective and promising inhibitors. Furthermore, when 4E-BP1 is hyperphosphorylated and dissociated from eIF4E, 4EGI-1 replaces 4E-BP1, hindering the eIF4G/eIF4E interaction. Thus, 4EGI-1 reinforces the translation inhibition function of 4E-BP1, providing an adjunctive tumor-suppressive role [59]. 4EGI-1 activity is also investigated in mouse model of ASD with beneficial effects [23].

Peptides and peptidomimetics are an emerging class of molecules able to modulate and inhibit a wide range of protein–protein interactions (PPIs) and cell membrane morphology. Their stability, specificity and low toxicity make peptidomimetics more suitable for therapeutic application than small compounds [171–174]. An example of this class is a fusion peptide containing residues 49–68 of 4E-BP1 and an analogue of gonadotropin-releasing hormone (GnRH) to target its receptor, widely overexpressed in ovarian and other endocrine tumors [175]. Notwithstanding their potential, there are limitations to the use of peptides, such as low secondary structural conformation in solution and poor permeability of the cellular membranes and physiological barriers (i.g. Blood–Brain Barrier). To overcome these limitations, chemical constraints can be added in specific positions of the peptides to increase the structural stability of the active conformation. Furthermore, peptidomimetics usually display improved metabolic stability and they are less subjected to proteolytic degradation compared to unmodified peptides [176]. Lama et al*.* described the development of second-generation hydrocarbon stapled-peptides in complex with eIF4E, showing increased binding kinetics and improved scaffold in terms of their degree of ordered helical structure [25, 35, 177].

Hence, peptides and peptidomimetics represent a new and attractive class of biomolecules, which will most likely play a central role in pharmacological applications for the development novel therapeutics.

## Conclusions and remarks

The eukaryotic translation initiation factor eIF4E plays a critical role in promoting the first step of the translation process. It is clearly established that dysregulation in the activity of eIF4E can be the primary cause of different types of diseases, such as cancer and neurodevelopmental disorders. Thus, it represents an attractive biological target for the development of drugs aimed to interfere with the interaction between eIF4E and its molecular partners eIF4G and 4E-BPs. The plethora of information accumulated in the last years on the structural features of these interactions, and their detailed analysis, is cardinal for the advancement of new therapeutic strategies. This structural information can be used in a near future to design novel and more specific eIF4E-targeting molecules with inhibitory potential, thus having an impact on the mRNA translation process.

## Data Availability

Not applicable.
